# 2-Hydr­oxy-5-nitro­benzaldehyde

**DOI:** 10.1107/S1600536809046807

**Published:** 2009-11-11

**Authors:** Hasan Tanak, Mustafa Macit, Metin Yavuz, Şamil Işık

**Affiliations:** a Department of Physics, Faculty of Arts & Science, Ondokuz Mayıs University, TR-55139 Kurupelit-Samsun, Turkey; b Department of Chemistry, Faculty of Arts & Science, Ondokuz Mayıs University, 55139 Samsun, Turkey

## Abstract

The title compound, C_7_H_5_NO_4_, is essentially planar, with a maximum deviation from the mean plane of 0.0116 (11) Å for the hydr­oxy O atom. The mol­ecular and crystal structure are stabilized by intra- and inter­molecular inter­actions. An intra­molecular O—H⋯O hydrogen bond generates a six-membered ring, producing an *S*(6) ring motif. The C—H⋯O inter­actions result in the formation of *C*(5) chains and *R*_2_^2^(8) rings forming an approximately planar network parallel to (10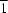). These planes are inter­connected through π–π inter­actions [centroid–centroid distance 3.582 (2) Å].

## Related literature

Nitro­aromatics are widely used as inter­mediates in explosives, dyestuffs, pesticides and organic synthesis, see: Yan *et al.* (2006 ▶). They occur in industrial wastes and as direct pollutants in the environment and are relatively soluble in water and detecta­ble in rivers, ponds and soil, see: Yan *et al.* (2006 ▶); Soojhawon *et al.* (2005 ▶). Aromatic compounds with multiple nitro substituents are known to be resistant to electrophilic attack by oxygenases, see: Halas *et al.* (1983 ▶). For comparison bond lengths and angles in related structures, see: Rizal *et al.* (2008 ▶); Garden *et al.* (2004 ▶). For hydrogen-bond motifs, see: Bernstein *et al.* (1995 ▶).
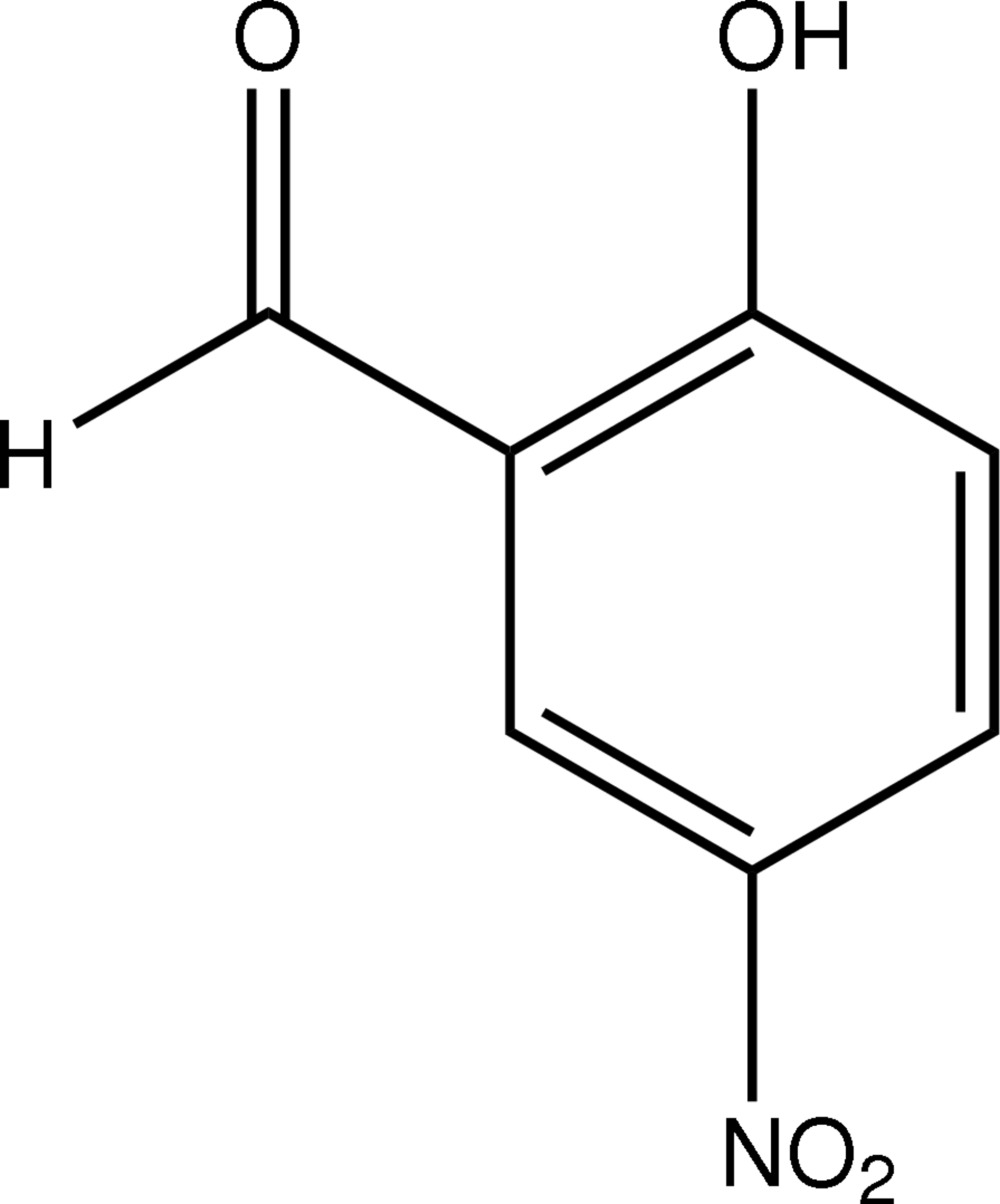


## Experimental

### 

#### Crystal data

C_7_H_5_NO_4_
*M* *_r_* = 167.12
Monoclinic, 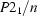

*a* = 7.2580 (17) Å
*b* = 8.3960 (13) Å
*c* = 11.704 (3) Å
β = 95.165 (18)°
*V* = 710.3 (3) Å^3^
*Z* = 4
Mo *K*α radiation
μ = 0.13 mm^−1^
*T* = 296 K
0.54 × 0.28 × 0.15 mm

#### Data collection

Stoe IPDS II diffractometer
Absorption correction: integration (*X-RED32*; Stoe & Cie, 2002 ▶) *T*_min_ = 0.979, *T*_max_ = 0.992
4345 measured reflections
1396 independent reflections
944 reflections with *I* > 2σ(*I*)
*R*_int_ = 0.062

#### Refinement

*R*[*F* ^2^ > 2σ(*F* ^2^)] = 0.050
*wR*(*F* ^2^) = 0.119
*S* = 1.06
1396 reflections
112 parameters
H atoms treated by a mixture of independent and constrained refinement
Δρ_max_ = 0.16 e Å^−3^
Δρ_min_ = −0.15 e Å^−3^

### 

Data collection: *X-AREA* (Stoe & Cie, 2002 ▶); cell refinement: *X-AREA*; data reduction: *X-RED32* (Stoe & Cie, 2002 ▶); program(s) used to solve structure: *SHELXS97* (Sheldrick, 2008 ▶); program(s) used to refine structure: *SHELXL97* (Sheldrick, 2008 ▶); molecular graphics: *ORTEP-3 for Windows* (Farrugia, 1997 ▶) and *PLATON* (Spek, 2009 ▶); software used to prepare material for publication: *WinGX* (Farrugia, 1999 ▶).

## Supplementary Material

Crystal structure: contains datablocks I, global. DOI: 10.1107/S1600536809046807/dn2510sup1.cif

Structure factors: contains datablocks I. DOI: 10.1107/S1600536809046807/dn2510Isup2.hkl

Additional supplementary materials:  crystallographic information; 3D view; checkCIF report

## Figures and Tables

**Table 1:** Hydrogen-bond geometry (Å, °)

*D*—H⋯*A*	*D*—H	H⋯*A*	*D*⋯*A*	*D*—H⋯*A*
C2—H2⋯O4^i^	0.93	2.50	3.427 (3)	175
C6—H6⋯O3^ii^	0.93	2.53	3.433 (3)	163
C7—H7⋯O2^iii^	0.93	2.50	3.176 (3)	130
O3—H3*A*⋯O4	0.93 (3)	1.73 (3)	2.613 (3)	157 (3)

Symmetry codes: (i) 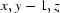; (ii) 
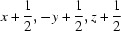
; (iii) 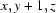.

**Fig. 1.**
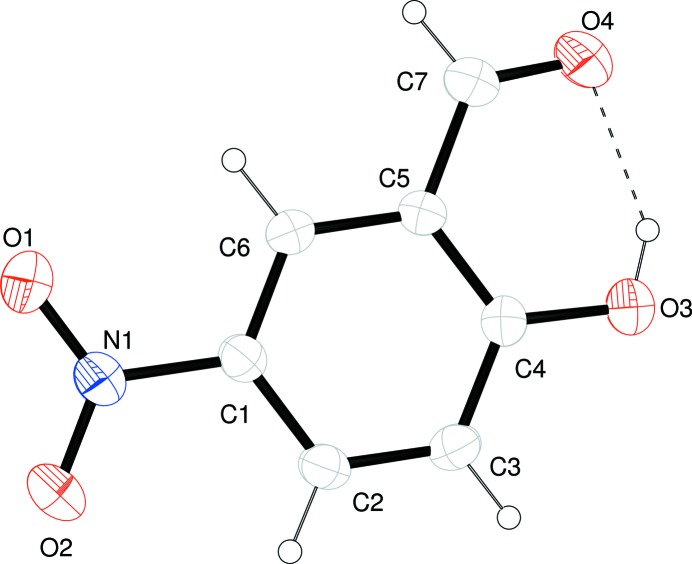
The molecular structure of the title compound, showing the atom-numbering scheme and 50% probability diplacement ellipsoids. H atoms are represented as small spheres of arbitrary radii.

**Fig. 2.**
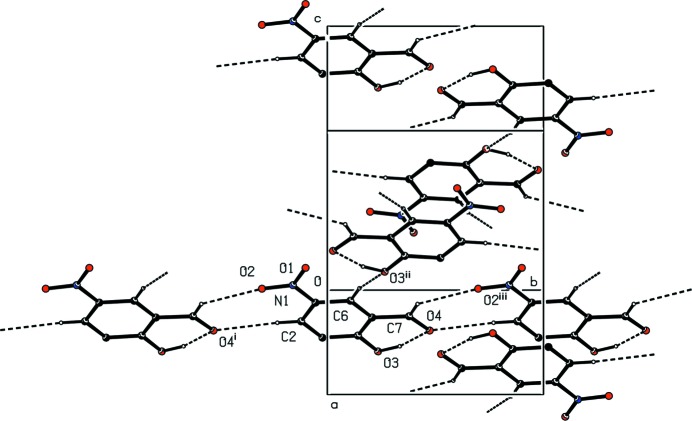
Partial packing view showing the formation of C-H···O hydrogen bonds represented as dashed lines. H atoms not involved in hydrogen bondings have been omitted for clarity. [Symmetry codes: (i) x, y-1, z; (ii) x+1/2, -y+1/2, z+1/2; (iii) x, y+1, z ]

## supplementary crystallographic information

### Comment

Nitroaromatics are widely used either as materials or as intermediates in explosives, dyestuffs, pesticides and organic synthesis (Yan *et al.*, 2006). Nitroaromatics occur as industrial wastes and direct pollutants in the environment, and are relatively soluble in water and detectable in rivers, ponds and soil (Yan *et al.*, 2006; Soojhawon *et al.*, 2005). Morover, aromatic compounds with multiple nitro substituents are known to be resistant to electrophilic attack by oxygenases (Halas *et al.*, 1983).

In the title compound (I, Fig. 1), the molecule is essentially planar with a maximum deviation from the mean plane of 0.0116 (11) Å for atom O3. The bond lengths and angles in (I) have normal values, and are comparable with those in the related structures (Rizal *et al.*, 2008; Garden *et al.*, 2004). The dihedral angle between the aromatic ring and the nitro group is 3.83 (3)°.

An intramolecular O3-H33···O4 interaction (Table 1, and Fig. 1) generates an S(6) ring motif (Bernstein *et al.*, 1995). In the crystal structure, the molecules are linked by intermolecular C2-H2···O4, C6-H6···O3 and C7-H7···O7 interactions into a three-dimensional framework. The C-H···O interactions result in the formation of C(5) chain but also R_2_^2^(8) ring forming an approximately planar network parallel to the (1 0 -1) plane (Fig. 2). These planes are interconnected through π-π interaction which occurs between Cg1 (the centroid of the C1-C6 ring) and its symmetry equivalent at (-x,-y,-z), with a centroid-to-centroid distance of 3.582 (2) Å, a plane-to-plane separation of 3.367 (1) Å and a slippage of 1.22 Å.

### Experimental

The commercially available compound (Acros Organics) was recrystallized from ethanol.

### Refinement

C-bound H atoms were positioned geometrically and refined using a riding model, with C—H = 0.93 Å and *U*_iso_(H) = 1.2*U*_eq_(C). The position of the H3A atom was obtained from a difference map of the electron density in the unit-cell and its coordinates were refined freely with *U*_iso_(H) = 1.5*U*_eq_(O) .

### Crystal data

**Table:**

C_7_H_5_NO_4_	*F*(000) = 344
*M**_r_* = 167.12	*D*_x_ = 1.563 Mg m^−^^3^
Monoclinic, *P*2_1_/*n*	Mo *K*α radiation, λ = 0.71073 Å
Hall symbol: -P 2yn	Cell parameters from 8368 reflections
*a* = 7.2580 (17) Å	θ = 1.8–27.3°
*b* = 8.3960 (13) Å	µ = 0.13 mm^−^^1^
*c* = 11.704 (3) Å	*T* = 296 K
β = 95.165 (18)°	Prism., red
*V* = 710.3 (3) Å^3^	0.54 × 0.28 × 0.15 mm
*Z* = 4	

### Data collection

**Table:**

Stoe IPDS II diffractometer	1396 independent reflections
Radiation source: fine-focus sealed tube	944 reflections with *I* > 2σ(*I*)
graphite	*R*_int_ = 0.062
Detector resolution: 6.67 pixels mm^-1^	θ_max_ = 26.0°, θ_min_ = 3.0°
rotation method scans	*h* = −8→8
Absorption correction: integration (*X-RED32*; Stoe & Cie, 2002)	*k* = −10→10
*T*_min_ = 0.979, *T*_max_ = 0.992	*l* = −14→14
4345 measured reflections	

### Refinement

**Table:**

Refinement on *F*^2^	Primary atom site location: structure-invariant direct methods
Least-squares matrix: full	Secondary atom site location: difference Fourier map
*R*[*F*^2^ > 2σ(*F*^2^)] = 0.050	Hydrogen site location: inferred from neighbouring sites
*wR*(*F*^2^) = 0.119	H atoms treated by a mixture of independent and constrained refinement
*S* = 1.06	*w* = 1/[σ^2^(*F*_o_^2^) + (0.05*P*)^2^ + 0.0581*P*] where *P* = (*F*_o_^2^ + 2*F*_c_^2^)/3
1396 reflections	(Δ/σ)_max_ < 0.001
112 parameters	Δρ_max_ = 0.16 e Å^−^^3^
0 restraints	Δρ_min_ = −0.15 e Å^−^^3^

### Special details

**Table:**

Experimental. 168 frames, detector distance = 120 mm
Geometry. All esds (except the esd in the dihedral angle between two l.s. planes) are estimated using the full covariance matrix. The cell esds are taken into account individually in the estimation of esds in distances, angles and torsion angles; correlations between esds in cell parameters are only used when they are defined by crystal symmetry. An approximate (isotropic) treatment of cell esds is used for estimating esds involving l.s. planes.
Refinement. Refinement of *F*^2^ against ALL reflections. The weighted *R*-factor *wR* and goodness of fit *S* are based on *F*^2^, conventional *R*-factors *R* are based on *F*, with *F* set to zero for negative *F*^2^. The threshold expression of *F*^2^ > σ(*F*^2^) is used only for calculating *R*-factors(gt) *etc*. and is not relevant to the choice of reflections for refinement. *R*-factors based on *F*^2^ are statistically about twice as large as those based on *F*, and *R*- factors based on ALL data will be even larger.

### Fractional atomic coordinates and isotropic or equivalent isotropic displacement parameters (Å^2^)

**Table:**

	*x*	*y*	*z*	*U*_iso_*/*U*_eq_	
O1	0.4340 (3)	−0.1006 (2)	0.25586 (14)	0.0788 (6)	
O2	0.3658 (3)	−0.3022 (2)	0.15003 (16)	0.0819 (6)	
O3	0.1019 (3)	0.2346 (2)	−0.19209 (14)	0.0702 (6)	
H3A	0.130 (4)	0.336 (4)	−0.162 (3)	0.105*	
O4	0.2251 (3)	0.4736 (2)	−0.06415 (16)	0.0895 (7)	
N1	0.3709 (3)	−0.1591 (2)	0.16564 (16)	0.0524 (5)	
C1	0.3019 (3)	−0.0545 (2)	0.07198 (17)	0.0425 (5)	
C2	0.2232 (3)	−0.1209 (2)	−0.03011 (17)	0.0459 (5)	
H2	0.2154	−0.2309	−0.0385	0.055*	
C3	0.1574 (3)	−0.0228 (3)	−0.11799 (18)	0.0494 (5)	
H3	0.1037	−0.0659	−0.1863	0.059*	
C4	0.1711 (3)	0.1417 (2)	−0.10464 (18)	0.0471 (5)	
C5	0.2551 (3)	0.2073 (2)	−0.00295 (17)	0.0437 (5)	
C6	0.3190 (3)	0.1067 (2)	0.08585 (17)	0.0431 (5)	
H6	0.3730	0.1485	0.1544	0.052*	
C7	0.2771 (3)	0.3784 (3)	0.0104 (2)	0.0631 (7)	
H7	0.3338	0.4172	0.0792	0.076*	

### Atomic displacement parameters (Å^2^)

**Table:**

	*U*^11^	*U*^22^	*U*^33^	*U*^12^	*U*^13^	*U*^23^
O1	0.1123 (15)	0.0673 (12)	0.0522 (10)	−0.0024 (11)	−0.0171 (10)	0.0069 (9)
O2	0.1202 (16)	0.0391 (10)	0.0829 (13)	0.0026 (9)	−0.0101 (11)	0.0101 (8)
O3	0.0914 (13)	0.0556 (11)	0.0590 (11)	0.0048 (9)	−0.0195 (9)	0.0061 (8)
O4	0.1338 (17)	0.0423 (10)	0.0871 (14)	0.0022 (11)	−0.0199 (12)	0.0094 (9)
N1	0.0593 (11)	0.0433 (12)	0.0547 (11)	−0.0005 (9)	0.0047 (9)	0.0071 (9)
C1	0.0418 (11)	0.0382 (12)	0.0475 (12)	−0.0002 (9)	0.0041 (9)	0.0023 (9)
C2	0.0486 (12)	0.0355 (10)	0.0535 (12)	−0.0007 (9)	0.0046 (9)	−0.0025 (9)
C3	0.0526 (12)	0.0492 (13)	0.0453 (12)	−0.0032 (10)	−0.0013 (9)	−0.0086 (10)
C4	0.0463 (11)	0.0458 (13)	0.0480 (12)	0.0029 (9)	−0.0018 (9)	0.0028 (10)
C5	0.0468 (11)	0.0366 (11)	0.0470 (12)	−0.0014 (9)	0.0007 (9)	−0.0008 (9)
C6	0.0457 (11)	0.0418 (12)	0.0413 (11)	−0.0031 (9)	0.0009 (9)	−0.0049 (9)
C7	0.0805 (17)	0.0424 (13)	0.0643 (15)	−0.0020 (12)	−0.0045 (12)	−0.0008 (12)

### Geometric parameters (Å, °)

**Table:**

O1—N1	1.216 (2)	C2—H2	0.9300
O2—N1	1.215 (2)	C3—C4	1.392 (3)
O3—C4	1.348 (2)	C3—H3	0.9300
O3—H3A	0.93 (3)	C4—C5	1.401 (3)
O4—C7	1.218 (3)	C5—C6	1.386 (3)
N1—C1	1.458 (3)	C5—C7	1.452 (3)
C1—C6	1.367 (3)	C6—H6	0.9300
C1—C2	1.394 (3)	C7—H7	0.9300
C2—C3	1.370 (3)		
			
C4—O3—H3A	100.5 (19)	O3—C4—C3	118.12 (19)
O2—N1—O1	122.27 (19)	O3—C4—C5	121.43 (19)
O2—N1—C1	118.59 (19)	C3—C4—C5	120.45 (19)
O1—N1—C1	119.13 (18)	C6—C5—C4	119.19 (18)
C6—C1—C2	121.56 (19)	C6—C5—C7	119.77 (19)
C6—C1—N1	119.05 (18)	C4—C5—C7	121.04 (19)
C2—C1—N1	119.38 (18)	C1—C6—C5	119.59 (19)
C3—C2—C1	119.5 (2)	C1—C6—H6	120.2
C3—C2—H2	120.3	C5—C6—H6	120.2
C1—C2—H2	120.3	O4—C7—C5	123.3 (2)
C2—C3—C4	119.71 (19)	O4—C7—H7	118.4
C2—C3—H3	120.1	C5—C7—H7	118.4
C4—C3—H3	120.1		

### Hydrogen-bond geometry (Å, °)

**Table:**

*D*—H···*A*	*D*—H	H···*A*	*D*···*A*	*D*—H···*A*
C2—H2···O4^i^	0.93	2.50	3.427 (3)	175
C6—H6···O3^ii^	0.93	2.53	3.433 (3)	163
C7—H7···O2^iii^	0.93	2.50	3.176 (3)	130
O3—H3A···O4	0.93 (3)	1.73 (3)	2.613 (3)	157 (3)

Symmetry codes: (i) *x*, *y*−1, *z*; (ii) *x*+1/2, −*y*+1/2, *z*+1/2; (iii) *x*, *y*+1, *z*.
